# The Role of Contrast-Enhanced Ultrasound in Differentiating Splenic Tuberculosis From Splenic Lymphoma

**DOI:** 10.3389/fonc.2022.891815

**Published:** 2022-06-16

**Authors:** Wenzhi Zhang, Gaoyi Yang, Xu Zhang, Tu Ni

**Affiliations:** Department of Ultrasonography, Affiliated Hangzhou Chest Hospital, Zhejiang University School of Medicine (Integrated Chinese and Western Hospital of Zhejiang Province, Hangzhou Red Cross Hospital), Hangzhou, China

**Keywords:** contrast-enhanced ultrasound, splenic tuberculosis, splenic lymphoma, high-frequency ultrasound, CDFI

## Abstract

**Aim:**

To summarize the features of splenic tuberculosis and splenic lymphoma by contrast-enhanced ultrasound (CEUS) and examine the application of CEUS in differentiating splenic tuberculosis from splenic lymphoma.

**Methods:**

The ultrasound and CEUS manifestations of 30 cases of splenic tuberculosis and 19 cases of splenic lymphoma were retrospectively analyzed, and the number of lesions, degree of splenomegaly, internal echogenicity, color blood flow signal, and CEUS manifestations of the two diseases were statistically determined.

**Results:**

A significant difference was noted in the internal echogenicity between splenic tuberculosis and splenic lymphoma lesions, particularly the strip-shaped hyperechoic areas of the lesions. The ultrasound features of perisplenic, retroperitoneal, and superficial lymph node enlargement were found to overlap (p < 0.05). Splenic tuberculosis showed heterogeneous enhancement and non-enhancement, whereas lymphoma showed low enhancement and high enhancement, and CEUS findings were statistically significant in distinguishing both, p < 0.05.

**Conclusion:**

Splenic tuberculosis is characterized by a lack of blood supply, mostly heterogeneous enhancement, and non-enhancement noted in CEUS. Splenic lymphoma lesions are often characterized by a rich blood supply and homogeneous enhancement on CEUS. CEUS can help identify the microcirculation of lesions in both patients with splenic lymphoma and patients with splenic tuberculosis. Thus, CEUS has great application value.

## Introduction

The imaging findings of various focal splenic lesions overlap, and color Doppler imaging is not helpful in the identification of splenic lesions. Contrast-enhanced ultrasound (CEUS) is increasingly being used in the differential diagnosis of splenic focal lesions. Owing to the clinical manifestations and imaging similarities between splenic lymphoma and splenic tuberculosis, these two diseases can be misdiagnosed. This study aimed to illustrate the CEUS and routine ultrasound [two-dimensional ultrasound (2D US) and color Doppler flow image (CDFI)] manifestations of the two diseases and summarize the value of CEUS in the differential diagnosis of the two diseases.

## Materials and Methods

### Patients

This study was reviewed and approved by the medical ethics committee of Affiliated Hangzhou Chest Hospital of Zhejiang University, and patients provided informed consent. From March 2015 to April 2022, 30 cases of splenic tuberculosis and 19 cases of splenic lymphoma confirmed by pathology were examined by ultrasound and CEUS at the Affiliated Hangzhou Chest Hospital of Zhejiang University. The pathological data of the lesion in the spleen of the patients were obtained using a core needle biopsy. Patients who consented to US and CEUS, those with complete medical information, those with normal blood clotting, and those without severe cardiopulmonary dysfunction were included in the study. Patients who were contraindicated for CEUS were excluded from the study. Inclusion criteria included patients diagnosed with splenic lymphoma and splenic tuberculosis and patients who had positive spleen findings by ultrasound and CEUS examination. Exclusion criteria included calcified splenic tuberculosis and a lesion larger and protrude from the spleen and adhesion to surrounding tissues and viscera.

### Ultrasound and Contrast-Enhanced Ultrasound Examination

A Philips ultrasonic diagnostic instrument (iU22, Philips Healthcare, Bothell), a high-frequency linear array probe (L12–L5, frequency 5–12 MHz; L9–L3, frequency 3–9 MHz), and a convex array low-frequency probe (C5–C1, frequency 1–5 MHz) were used for patient examinations. The characteristics of routine ultrasound and CEUS were recorded for the lesions in the spleen such as the size and number of lesions, the degree of splenomegaly, the internal echo of lesions, and the internal color blood flow signal.

CEUS examination with low mechanical index (0.06) pulse reverse harmonic imaging and the second-generation sulfur hexafluoride microbubble ultrasound contrast agent SonoVue (Bracco SpA, Milan, Italy) were used for patient examination. The elbow vein was initially injected with 2.4 ml of SonoVue, which was followed by another injection of 5 ml of saline. Next, dynamic observation of the lesion in the spleen enhancement was conducted followed by continuous observation for 2 min.

The CEUS patterns of splenic tuberculosis are heterogeneous enhancement and non-enhancement. Heterogeneous enhancement can be divided into septal enhancement and marginal enhancement. The CEUS pattern of splenic lymphoma is homogeneous enhancement. The number of cases in which the lesions in the spleen could not be identified using routine ultrasound or the presence of lesions was uncertain but the lesions in the spleen were identified using CEUS was recorded.

To reduce subjective error, both routine ultrasound and CEUS enhancement were performed by two attending physicians with 5 years of experience in ultrasound diagnosis, who were blinded to the pathological results. The data were independently diagnosed and analyzed by two sonographers, followed by a discussion to unify the results.

### Statistical Analysis

The data were analyzed by the statistical software SPSS 23.0 (IBM, USA). Counting data for the difference between routine ultrasound and CEUS of the spleen lesions of different pathological types were analyzed by *χ*^2^ test and Fisher’s exact test. A p value <0.05 was statistically significant.

## Results

### Patients and Study Design

The splenic tuberculosis group (30 cases) included 12 men and 18 women who were 18–68 years old with an average age of 34.17 ± 3.344 years.

The splenic lymphoma group (19 cases) included 8 men and 11 women who were 31–73 years old with an average age of 40.022 ± 6.712 years. The mean age at the onset of splenic tuberculosis was >40 years, whereas the mean age at the onset of splenic lymphoma was <40 years. There were two patients with splenic tuberculosis and two patients with splenic lymphoma who were over the age of 60 years. The age distribution is shown in **Table 1**.

**Table 1:** Comparison of splenic tuberculosis and splenic lymphoma by 2D and color Doppler ultrasound.

		Splenic tuberculosis (30 cases)	Splenic lymphoma (19 cases)	χ2	P
Gender	Male	12	8		
	Female	18	11	0.021	0.884
Age	<40 years	22	2		
	>41 years	8	17	18.363	0
	Very low echogenicity	0	5	8.792	0.006
	Low echogenicity	13	13	2.94	0.142
Internal echogenicity	Mixed echogenicity	17	1	13.226	0.001
Strip hyperechogenicity in the lesion	Yes	0	6	0	0.002
	No	30	13		
Nodular conglomeration	Yes	5	8	3.862	0.095
	No	25	11		
Low-frequency ultrasound shows nodules	Yes	26	17	0	1
	No	4	2		
Lymphadenopathy	Yes	5	14	15.93	0
	No	25	5		

Lymphadenopathy includes: Accompanied by perisplenic, retroperitoneal, superficial lymph node swelling.

Twenty-four cases of splenic tuberculosis were cured after antituberculosis treatment, and six cases were confirmed by needle biopsy. All patients with splenic lymphoma were pathologically confirmed by biopsy, and no significant abdominal hemorrhage was noted after biopsy.

### Ultrasound Examination

Routine ultrasound revealed that all of the patients in both groups had multiple lesions, with the exception of one case of splenic tuberculosis in which only a single lesion was noted. Mild splenomegaly was observed in 8 cases of splenic tuberculosis. Splenomegaly was observed in 11 cases of lymphoma, including mild splenomegaly in 7 cases and moderate splenomegaly in 4 cases. CDFI ultrasonography indicated that there was no obvious color blood flow signal in the splenic tuberculosis lesion, dotted color blood flow signal in the peripheral lesion, and striped color blood flow signal in the splenic lymphoma lesion. In four cases of splenic tuberculosis and two cases of splenic lymphoma, the lesions could not be detected by low-frequency ultrasound; in four cases of splenic tuberculosis, the lesions could be clearly visualized by high-frequency ultrasound. Of the two patients with splenic lymphoma, one case showed an uneven splenic echo by high-frequency ultrasound and the other case showed no lesion.

Internal echo of the lesion, conglomeration of the lesion, and abdominal, retroperitoneal, or superficial lymph node enlargement are shown in **Table 1**.

### Contrast-Enhanced Ultrasound Examination

CEUS of patients with splenic tuberculosis showed homogeneous enhancement (2/30, 6.7%), heterogeneous enhancement (19/30, 63.3%), and no enhancement (9/30, 30.0%), with most cases showing low enhancement. Among these cases, heterogeneous enhancement was divided into the septal enhanced type (4/19, 21.1%) and the marginal enhanced type (15/19, 78.9%).

For patients with splenic lymphoma, CEUS showed homogeneous enhancement (17/19, 89.5%), with 52.6% showing low enhancement (10/19) and 47.4% showing high enhancement (9/19).

CEUS showed that the probability of 2D US failing to detect the lesions in the spleen was low, which was not statistically significant because of the small number of cases. The results of CEUS for the two diseases are shown in **Table 2**.

**Table 2:** Comparison of splenic tuberculosis and splenic lymphoma by CEUS.

CEUS findings		Splenic tuberculosis (30 cases)	Splenic lymphoma (19 cases)	*χ*^2^	p
Enhanced mode	Homogeneous enhancement	2	17	33.599	0.000
	Heterogeneous enhancement	19	1	16.239	0.000
	No enhancement	9	1	2.992	0.084
Enhanced strength	High enhancement	0	9	14.392	0.000
	Low enhancement	30	10		
Lesions that were not detected by US were identified by CEUS	Yes	0	2	0.000	0.145
	No	30	17		

CEUS, contrast-enhanced ultrasound; US, ultrasound.

## Discussion

The spleen is involved in lymphomas. Approximately 30%–40% of cases of systemic Hodgkin’s disease and non-Hodgkin’s lymphoma involve the spleen. The incidence of primary splenic lymphoma is much lower than that of secondary splenic lymphoma, accounting for less than 1% of all splenic lymphoma cases (1). Splenic lymphoma occurs in nearly all age groups and does not exhibit predisposition toward a particular gender. Considering the histological similarity between primary and secondary lymphomas of the spleen, the imaging features are identical (1). The imaging manifestations of various focal splenic lesions are found to overlap, and CDFI has a low value in differentiating splenic lesions. CEUS has been increasingly applied in the diagnosis and differential diagnosis of focal splenic lesions (1).

Splenic tuberculosis is divided into primary and secondary splenic tuberculosis. The incidence of primary splenic tuberculosis is rare. Although Winternitz (2) classified splenic tuberculosis into primary and secondary types in 1912, some researchers have demonstrated that splenic tuberculosis is a *Mycobacterium tuberculosis* infection secondary to other organs. In this study, the age at the onset of splenic tuberculosis was <40 years, whereas the age at the onset of splenic lymphoma was 41–60 years. On entering the body, *M. tuberculosis* is consumed by macrophages, after 2–4 weeks to produce a cell-mediated immune response and late onset of allergy, the immune response mainly make lymphocyte sensitization, macrophages hyperplasia, characteristic lesions limited, and generate tuberculous granuloma, late onset of allergy is caused cells caseous necrosis, tissue damage, It can be combined with splenic vein thrombosis (3, 4). The ultrasonographic manifestations of splenic tuberculosis vary in the different stages of the disease, and there is no unified standard for the classification of splenic tuberculosis using ultrasound in domestic and foreign literature at present. There are three types of routine ultrasound manifestation of splenic tuberculosis: hypoechoic, mixed echoic, and strong echoic. Because the hyperechoic type is mostly the ultrasound manifestation after healing, it is extremely different from the common hypoechoic manifestation of splenic lymphoma; therefore, it has not been included in the study scope.

**Figure 1:**
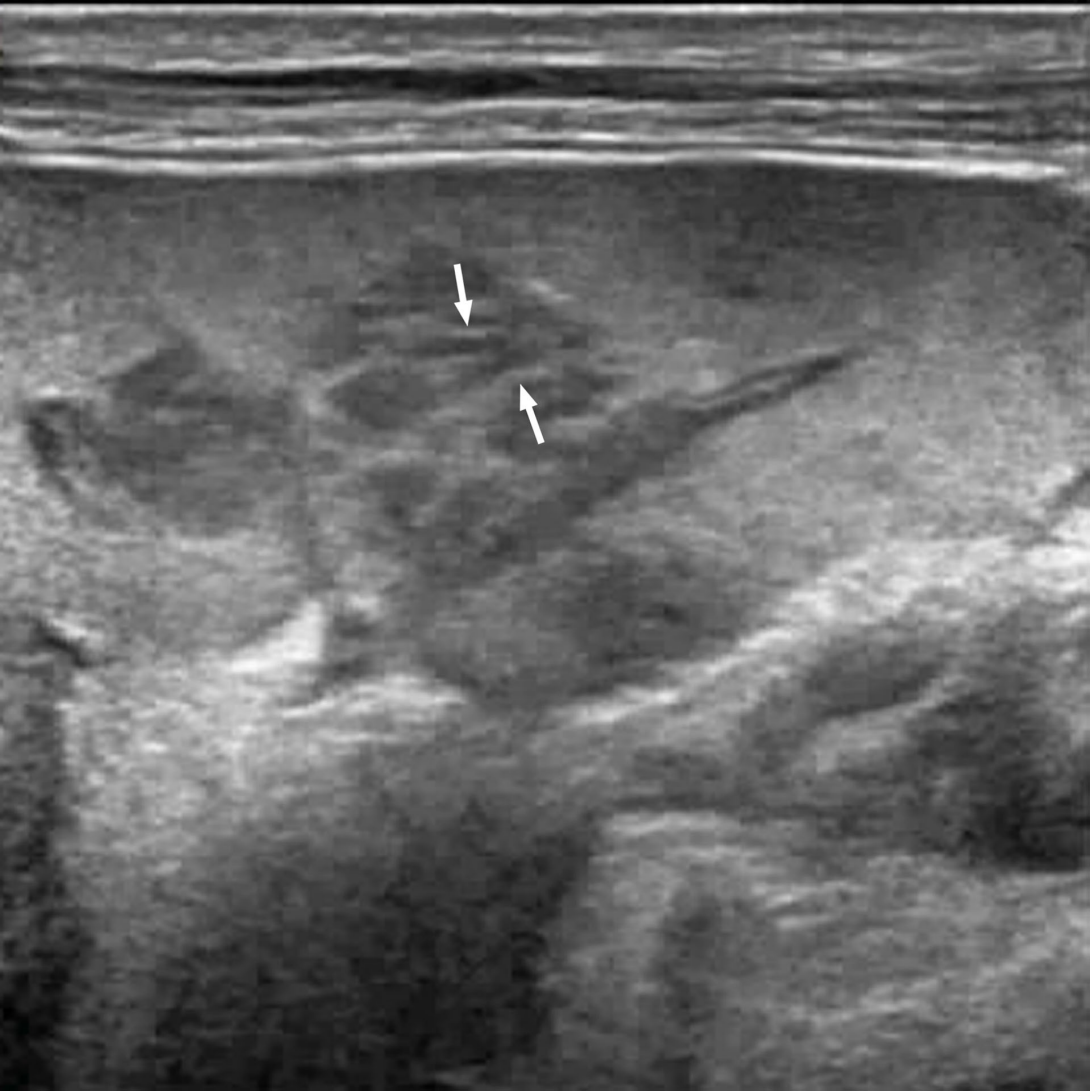
A 67-year-old man with splenic lymphoma. Multiple hypoechoic nodules are visible in the splenic region. Some of the nodules are fused with each other. Striations in the hyperechoic areas are observed in the nodule (arrow).

In this study, cases of splenic tuberculosis and splenic lymphoma were mainly characterized by multiple nodules in the spleen; thus, the number of nodules had no obvious specificity toward distinguishing the two diseases. Splenic tuberculosis focus is low echogenicity and mixed echogenicity and no extremely low echogenicity lesions, and splenic lymphoma is given priority if with extremely low echogenicity and low echogenicity lesions. Therefore, extremely low echogenicity is an important reference of lymphoma with a splenic tuberculosis differential indicator, but extremely low echogenicity in this study is not a very high incidence in lymphoma, about 26.31% (5/19). In this study, ultrasound showed the presence of strip-shaped hyperechoic areas in lymphoma lesions but not in splenic tuberculosis lesions. We believe that strip-shaped hyperechoic areas noted in ultrasound are characteristic of lymphoma lesions (**Figure 1**). Both diseases are there will be accompanied by spleen weeks, retroperitoneal, superficial lymph node enlargement, in areas such as lymphoma in this study are more likely to appear aforementioned place swollen lymph nodes, in patients with cervical lymph node enlargement, can be on the neck lymph node biopsy, can cause of intumescent lymph node of diagnosis, and can assist in the diagnosis of splenic lymphoma, and feasible to percutaneous splenic biopsy diagnosed, Can avoid the pain of cesarean section.

Both splenic tuberculosis and splenic lymphoma lesions can be fused. In this study, splenic lymphoma was found to be more easily fused than splenic tuberculosis; the incidence of splenic tuberculosis conglomeration was 20% (5/30) and that of splenic lymphoma conglomeration was 42.10% (8/19). Therefore, mutual conglomeration between lesions was not the specific ultrasonic manifestation of one of the two diseases. Its value in terms of differential diagnosis remains to be further confirmed. However, in patients suspected of having lymphoma, the immune function is normal; the mutual conglomeration between lesions, the strip-shaped hyperechoic areas within the lesions, the rich blood supply to the lesions appear simultaneously; and the perisplenic, retroperitoneal, and superficial lymph node enlargement should be especially considered in cases of lymphoma (5).

In this study, there were four cases of miliary splenic tuberculosis and two cases of lymphoma. No obvious nodules or uneven echoes in the splenic region were found using low-frequency ultrasound. However, a high-frequency probe of 5–12 MHz was used to achieve satisfactory results, indicating the presence of low-echo nodules in the splenic region. In this study, six cases of splenic lymphoma lesions showed strip-shaped hyperechoic areas. In four cases, high-frequency ultrasound showed strip-shaped hyperechoic areas more clearly than low-frequency ultrasound. For patients without obesity having intrasplenic lesions near the body surface, high-frequency ultrasound can be used to observe the internal structure of lesions more clearly, and high-frequency ultrasound examination of the spleen can reduce cases of misdiagnosis (6). High-frequency ultrasonic-assisted low-frequency ultrasound examination may be a good method for the examination of the spleen.

**Figure 2:**
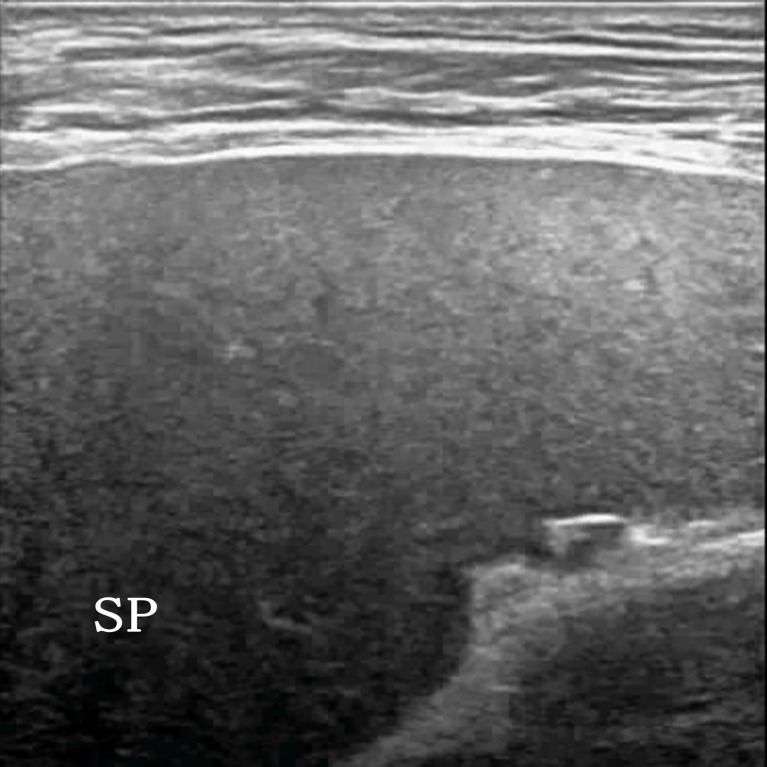
A 54-year-old man clinically suspected of having splenic lymphoma. Two-dimensional ultrasound (2D US) showed uniform splenic echogenicity.

**Figure 3:**
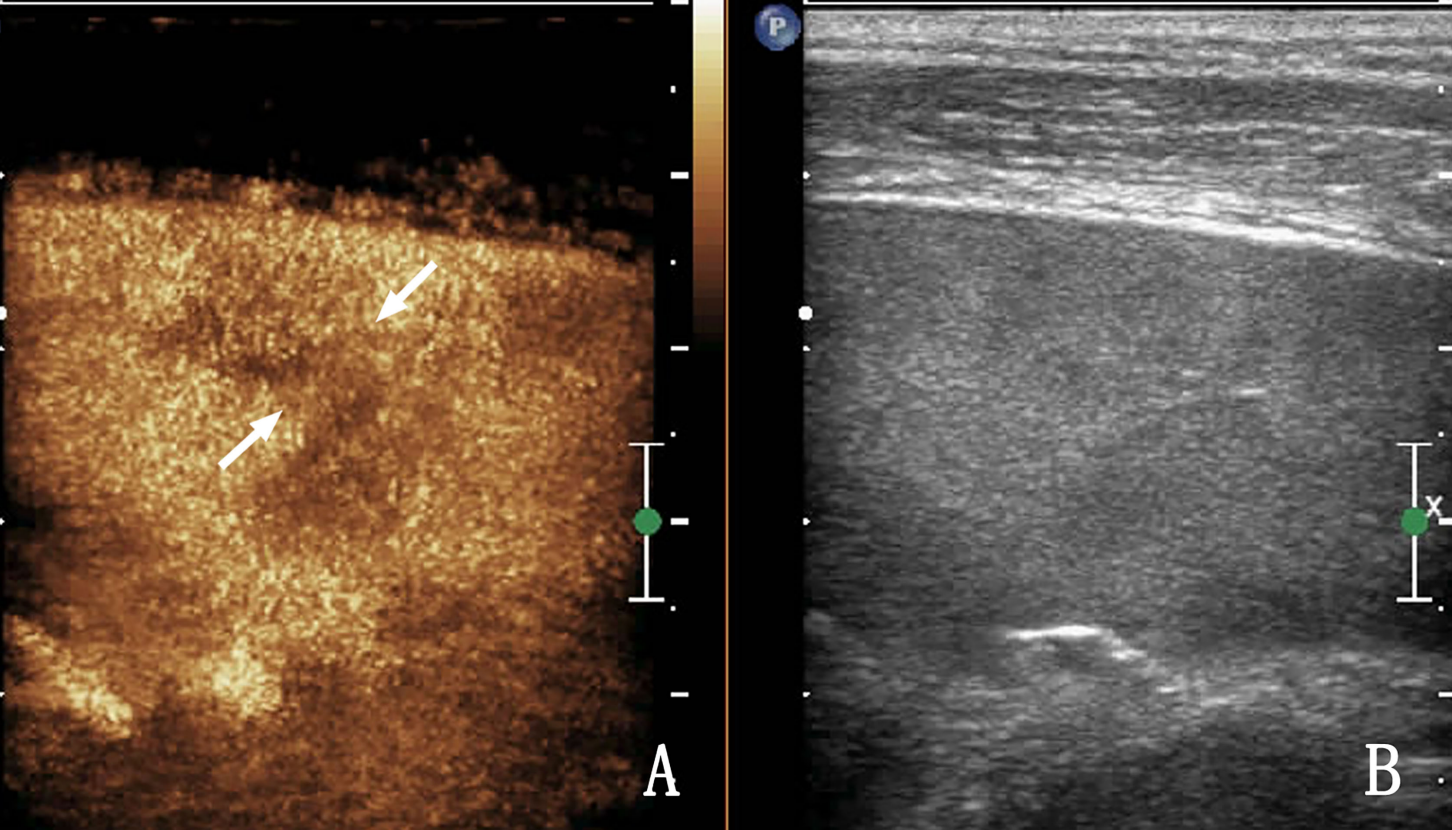
The same patient as that in **Figure 2**. **(A)** Contrast-enhanced ultrasound (CEUS) shows a low enhancement area in the spleen, and nodules appear to be conglomerated with each other (arrow) without obvious capsule enhancement. **(B)** Shows a two-dimensional ultrasound (2D US) image. Ultrasound-guided biopsy of the low enhancement area pathologically confirmed splenic lymphoma.

The sensitivity of 2D US in terms of the diagnosis of splenic lymphoma involvement is only 54%, with a specificity of 100% (7); the display rate is very high, but there are also cases that did not show splenic lesions in 2D US, and CEUS imaging showed clear splenic lesions (**Figure 2**, **Figure 3**). The incidence rate was only 4.08% (2/49) in this study for splenic lymphoma. Because of the small number of cases, no statistical significance was noted. Although the incidence is low, but given our lesson is very deep, when clinical prompt splenomegaly suspected splenic lymphoma, when found 2D US not shown significant lesions, show only the spleen uneven echogenicity, CEUS very be necessary, once the CEUS prompted low enhanced nodule, to alert to the possibility of splenic lymphoma, it is best to do biopsy diagnosis. In patients suspected of having splenic lymphoma wherein no obvious lesions are noted in the spleen area or uneven echoes are noted in the spleen area, CEUS can be used to evaluate the nodules within its blood supply from the state, such as homogeneous enhancement in both low enhancement and high enhancement should consider splenic tumor, splenic lymphoma is in the spleen of primary malignant tumor of the first (8). Therefore, lymphomas can be considered first if ultrasound and CEUS suggest the presence of malignant tumor of the spleen without the involvement of extrasplenic organs.

**Figure 4:**
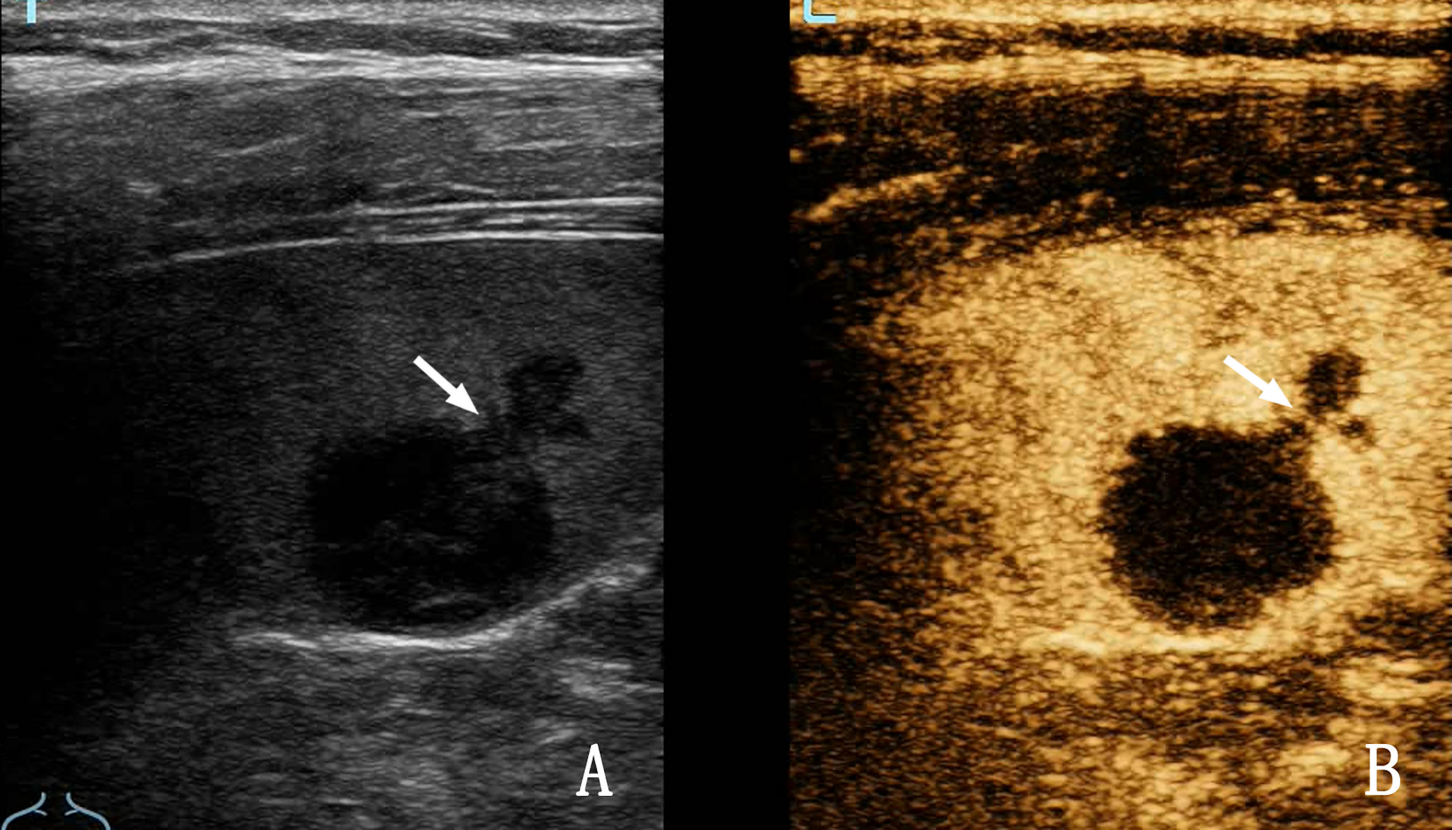
A 32-year-old woman with splenic tuberculosis. **(A)** Two-dimensional ultrasound (2D US) shows multiple nodules in the splenic region. **(B)** Contrast-enhanced ultrasound (CEUS) shows that two nodules are conglomerated (arrows), both of which are non-enhanced.

**Figure 5:**
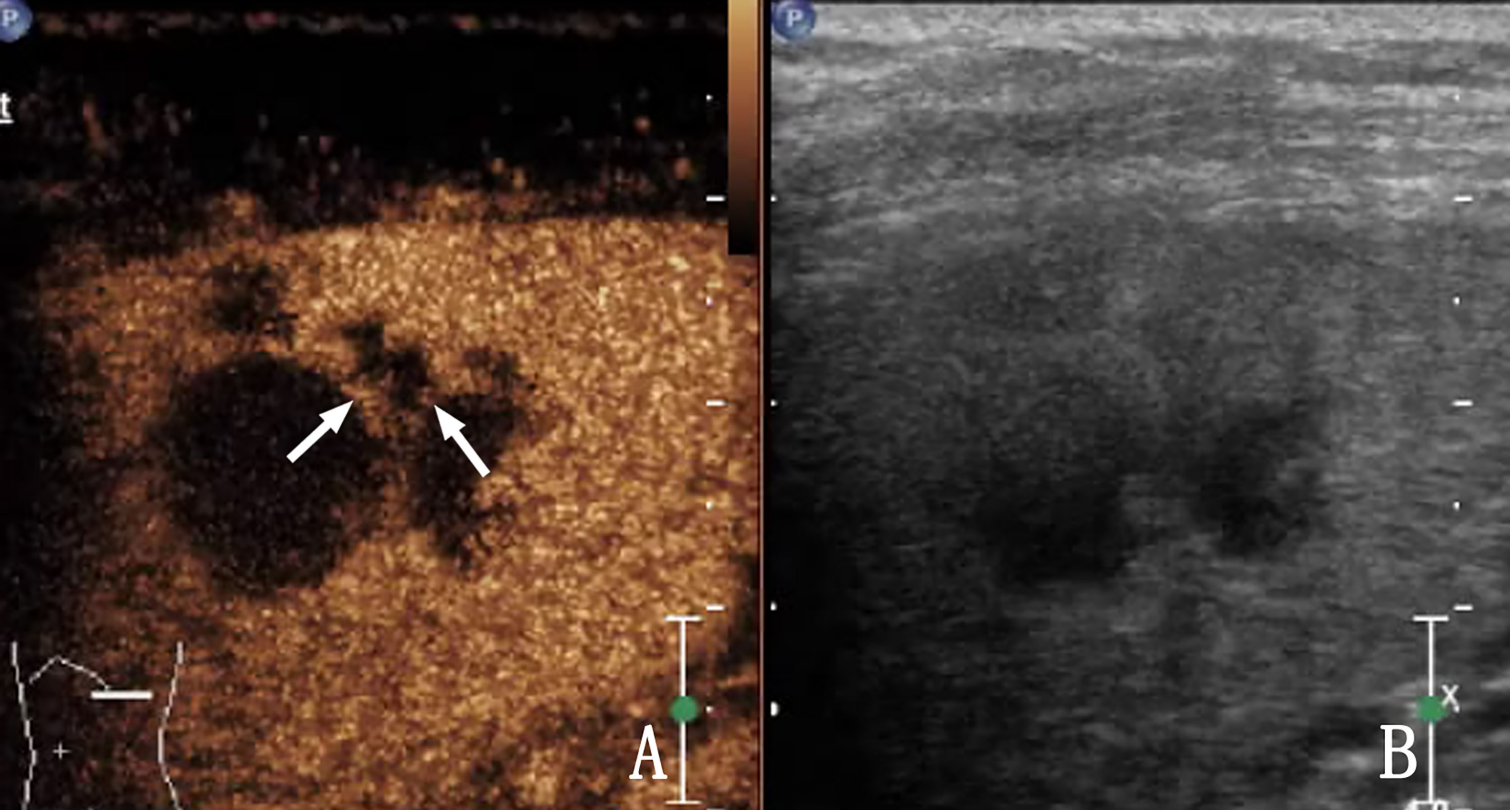
A 28-year-old woman with splenic tuberculosis. **(A)** Contrast-enhanced ultrasound (CEUS) shows heterogeneous enhancement of the lesion within the spleen, and septal enhancement (arrow) can be observed. **(B)** Shows a two-dimensional ultrasound (2D US) image.

In cases of splenic tuberculosis, 2D US did not show intrasplenic lesions; but clear occurred by CEUS is rarely, which may be associated with its pathological features. The typical pathological process of splenic tuberculosis is as follows: exudation, caseous transformation, proliferation, obvious acoustic impedance difference with the spleen parenchyma, and lack of blood supply in the spleen. The display rate of 2D US for splenic tuberculosis is already extremely high. CEUS has little value in improving the display rate of splenic tuberculosis, but it is superior to 2D US in evaluating the internal blood supply to the lesion (9–12). CEUS of splenic tuberculosis showed that the lesions were mostly non-enhanced (**Figure 4**) (13). Thin septal enhancement was observed in some heterogeneous enhanced lesions (**Figure 5**), and splenic lymphoma rare this sign, for splenic tuberculosis infectious disease, blood vessels are less damaging, remaining within the organization can exist in the spleen of lesions, and lymphoma rare necrosis, necrosis is more than spleen infarction, necrosis is more complete. We believe that septal enhancement within lesions is a crucial CEUS index for the differential diagnosis of splenic tuberculosis and splenic lymphoma. On the appearance of such signs, the possibility of lymphoma can be excluded. Unfortunately, the proportion of those with septal enhancement in splenic tuberculosis was not high (13.33%, 4/30) in this study.

## Conclusion

Splenic tuberculosis is characterized by a lack of blood supply, low echogenicity or mixed echogenicity on ultrasound, and mostly heterogeneous enhancement and non-enhancement noted in CEUS. Splenic lymphoma lesions are often characterized by a rich blood supply and homogeneous enhancement on CEUS. CEUS can help identify lesions in the microcirculation and evaluate the characteristics of the blood supply in lesions in both patients with splenic lymphoma and patients with splenic tuberculosis. Thus, CEUS modality has great application value.

## Data Availability Statement

The datasets presented in this study can be found in online repositories. The names of the repository/repositories and accession number(s) can be found in the article/supplementary material.

## Ethics Statement

The studies involving human participants were reviewed and approved by the Medical Ethics Committee of Affiliated Hangzhou Chest Hospital of Zhejiang University. The patients/participants provided their written informed consent to participate in this study.

## Author Contributions

WZ was responsible for project administration, methodology, data creation, and writing—review and editing. GY, XZ, and TN were responsible for data curation and paper revision. All authors contributed to the article and approved the submitted version.

## Funding

This work was supported by the Hangzhou Agriculture and Social Development Research Project (20190101A09).

## Conflict of Interest

The authors declare that the research was conducted in the absence of any commercial or financial relationships that could be construed as a potential conflict of interest.

## Publisher’s Note

All claims expressed in this article are solely those of the authors and do not necessarily represent those of their affiliated organizations, or those of the publisher, the editors and the reviewers. Any product that may be evaluated in this article, or claim that may be made by its manufacturer, is not guaranteed or endorsed by the publisher.

## References

[1] Sutherland T Temple F Hennessy O Lee WK. Contrast-Enhanced Ultrasound Features of Primary Splenic Lymphoma. J Clin Ultrasound (2010) 38:317–9. doi: 10.1002/jcu.20699 20544868

[2] Khadka M Pradhan R. Isolated Splenic Cold Abscesses With Perisplenic Extension: Treated Successfully Without Splenectomy. Case Rep Gastrointest Med (2017) 2017:9864543. doi: 10.1155/2017/9864543 28912985 PMC5585634

[3] Natarajan V John K Jose D Kalaiselvan P Das AK. Portal Venous Thrombosis-Disseminated Tuberculosis in Rheumatoid Arthritis. J Clin Diagn Res (2017) 11:OD08–10. doi: 10.7860/JCDR/2017/27640.10048 PMC5535419 28764227

[4] Jain D Verma K Jain P. Disseminated Tuberculosis Causing Isolated Splenic Vein Thrombosis and Multiple Splenic Abscesses. Oxf Med Case Rep (2014) 2014:107–9. doi: 10.1093/omcr/omu042 PMC4369988 25988047

[5] Dachman AH Buck JL Krishnan J Aguilera NS Buetow PC. Primary non-Hodgkin’s Splenic Lymphoma. Clin Radiol (1998) 53:137–42. doi: 10.1016/S0009-9260(98)80061-5 9502091

[6] Görg C Faoro C Bert T Tebbe J Neesse A Wilhelm C. Contrast Enhanced Ultrasound of Splenic Lymphoma Involvement. Eur J Radiol (2011) 80:169–74. doi: 10.1016/j.ejrad.2009.11.012 20005061

[7] Görg C Weide R Schwerk WB. Malignant Splenic Lymphoma: Sonographic Patterns, Diagnosis and Follow-Up. Clin Radiol (1997) 52:535–40. doi: 10.1016/S0009-9260(97)80331-5 9240707

[8] Bhatia K Sahdev A Reznek RH. Lymphoma of the Spleen. Semin Ultrasound CT MRI (2007) 28:12–20. doi: 10.1053/j.sult.2006.10.010 17366704

[9] Yang R Lu Q Xu J Huang J Gao B Zhang H. Value of Contrast-Enhanced Ultrasound in the Differential Diagnosis of Focal Splenic Lesions. Cancer Manag Res (2021) 13:2947–58. doi: 10.2147/CMAR.S300601 PMC8021137 33833578

[10] Schwarze V Lindner F Marschner C Negrão De Figueiredo G Rübenthaler J Clevert DA. Single-Center Study: The Diagnostic Performance of Contrast-Enhanced Ultrasound (Ceus) for Assessing Focal Splenic Lesions Compared to Ct and Mri. Clin Hemorheol Microcirc (2019) 73:65–71. doi: 10.3233/CH-199204 31561333

[11] Li XZ Song J Sun ZX Yang YY Lin YQ Wang H. Conventional Ultrasound and Contrast-Enhanced Ultrasound in the Diagnosis of Splenic Diseases. J Ultras J Ultrasound Med (2020) 39:1687–94. doi: 10.1002/jum.15291 32323353

[12] Zavariz JD Konstantatou E Deganello A Bosanac D Huang DY Sellars ME. Common and Uncommon Features of Focal Splenic Lesions on Contrast-Enhanced Ultrasound: A Pictorial Review. Radiol Bras (2017) 50:395–404. doi: 10.1590/0100-3984.2015.0209 29307931 PMC5746885

[13] Zhang Y Yu T Zhang W Yang G. Contrast-Enhanced Ultrasound Imaging Features of Focal Splenic Tuberculosis. Med Sci Monit (2021) 27:e932654. doi: 10.12659/MSM.932654 34526476 PMC8454255

